# Detachment of cell sheets from clinically ubiquitous cell culture vessels by ultrasonic vibration

**DOI:** 10.1038/s41598-020-66375-1

**Published:** 2020-06-11

**Authors:** Chikahiro Imashiro, Makoto Hirano, Takashi Morikura, Yuki Fukuma, Kiyoshi Ohnuma, Yuta Kurashina, Shogo Miyata, Kenjiro Takemura

**Affiliations:** 1 Department of Mechanical Engineering, Keio University, 3-14-1 Hiyoshi, Kohoku-ku, Yokohama, 223-8522 Japan; 2 Institute of Advanced Biomedical Engineering and Science, Tokyo Women’s Medical University, TWIns, 8-1 Kawada-cho, Shinjuku-ku, Tokyo, Japan; 3 Department of Pharmacy, Yasuda Women’s University, 6-13-1 Yasuhigashi, Asaminami-ku, Hiroshima, Japan; 4 School of Science for Open and Environmental Systems, Graduate School of Science and Technology, Keio University, 3-14-1 Hiyoshi, Kohoku-ku, Yokohama, 223-8522 Japan; 5 Department of Bioengineering, Nagaoka University of Technology, 1603-1 Kamitomioka, Nagaoka, Niigata, 940-2188 Japan; 6 Department of Science of Technology Innovation, Nagaoka University of Technology, 1603-1 Kamitomioka-cho, Nagaoka, Niigata, 940-2188 Japan; 7 Department of Materials Science and Engineering, School of Materials and Chemical Technology, Tokyo Institute of Technology, Yokohama, 226-8503 Japan

**Keywords:** Tissue engineering, Cell adhesion

## Abstract

Proteinases that digest the extracellular matrix are usually used to harvest cells from culture vessels in a general culture process, which lowers the initial adhesion rate in regenerative medicine. Cell sheet engineering is one of the most important technologies in this field, especially for transplantation, because fabricated cell sheets have rich extracellular matrixes providing strong initial adhesion. Current cell sheet fabrication relies on temperature-responsive polymer-coated dishes. Cells are cultured on such specialized dishes and subjected to low temperature. Thus, we developed a simple but versatile cell sheet fabrication method using ubiquitous culture dishes/flasks without any coating or temperature modulation. Confluent mouse myoblasts (C2C12 cell line) were exposed to ultrasonic vibration from underneath and detached as cell sheets from entire culture surfaces. Because of the absence of low temperature, cell metabolism was statically increased compared with the conventional method. Furthermore, viability, morphology, protein expression, and mRNA expression were normal. These analyses indicated no side effects of ultrasonic vibration exposure. Therefore, this novel method may become the standard for cell sheet fabrication. Our method can be easily conducted following a general culture procedure with a typical dish/flask, making cell sheets more accessible to medical experts.

## Introduction

Tissue engineering and regenerative medicine have been developed, in which cell sheet development is one of the major challenges^1,2^. It has been reported that cell sheet implantation improves therapeutic effects compared with implantation of dissociated cells^3,4^. For example, a myocardial cell sheet is an appropriate graft for regenerative medicine of the myocardium^5,6^. In addition, there are many possible treatments of any organ such as the cornea, cartilage, and esophagus^7–9^. However, in the field of tissue engineering, accumulated cell sheets are used to generate a 3-D tissue *in vitro*^10^.

Current cell sheet fabrication for practical use exclusively relies on a temperature-responsive culture dish with poly-N-isopropylacrylamide (pNIPAAm) coated on its culture surface. After reaching confluency on the pNIPAAm-coated dish surface, cells are subjected to a low temperature of 20 °C. The surface-bound pNIPAAm undergoes a reversible change from hydrophobic to hydrophilic upon lowering the temperature. As a result, the cell sheet detaches^11,12^. Because this cell sheet fabrication procedure does not use any proteinases that might degrade surface proteins, the fabricated cell sheet has a rich extracellular matrix (ECM) providing strong initial adhesion. In addition to the simplicity of handling a cell sheet compared with dissociated cells, this ECM-rich feature is highly effective for transplanting cultured cells.

However, the use of temperature-responsive polymer-coated culture dishes has two main problems. First, it requires a reduction in temperature to detach a cell sheet from a dish, resulting in a decrease of cell viability and variation of the functions of some delicate cells^13–15^. Second, pNIPAAm coating is indispensable. To coat pNIPAAm on a culture surface, electron beam or vapour phase polymerization equipment and facilities are needed^16^, which is technically difficult and costly. Although UpCell^®^-precoated temperature-responsive culture dishes are commercially available, this specialized cell culture dish is much more expensive than a general culture dish. In addition to using temperature-responsive culture dishes, ECM-rich cell sheets have been fabricated by other techniques such as using electro- and photo-responsive materials, modulation of medium, and infusing polymers^8,17–20^. These alternatives for detaching a cell sheet may overcome the two major problems of pNIPAAm-coated dishes. However, they still require specialized processing of a cell culture surface or chemicals that could be harmful to cells. Consequently, a pNIPAAm-coated dish is the only method to generate a clinically applicable cell sheet. Thus, a simple cell sheet fabrication method capable of generating an active and ECM-rich cell sheet is highly desired for the advancement of regenerative medicine.

Thus, the purpose of this study was to develop a simple technique to generate an active and ECM-rich cell sheet with a clinically ubiquitous cell culture dish/flask, which does not require temperature modulation, additional chemicals, or specialized technical skills. Our previous study showed that cells cultured on an ultrasonic transducer can be detached by the combination of ultrasonic vibration and temperature modulation without any enzymes^21^. In addition, cells detached by our method have much more intact membrane proteins than those detached by the conventional trypsin treatment. Moreover, the initial adhesion time of cells to a culture surface is much shorter for cells detached by our method compared with cells detached by trypsin^21^. In another previous report, the detachment trigger was identified as mechanical force generated by acoustic radiation^22^. Therefore, we investigated detachment of an ECM-rich cell sheet from the entire culture surface of clinically ubiquitous cell cultures vessel by ultrasonic vibration that is capable of detaching cells forcibly. We developed a cell sheet-detaching system, detached a cell sheet, and estimated the functionality of the cell sheet in this study. The efficiency of the developed method was demonstrated by comparison with the conventional method using a pNIPAAm-coated culture dish, which is the only method used practically. Mouse myoblasts (C2C12 cell line) were used as a model cell line in our study. The C2C12 cell line is widely used in cell engineering experiments, including cell sheet-detaching studies, because myoblast cell sheet transplantation is a significant regenerative therapy that has been reported previously^23–25^.

## Results

### Cell sheet detachment

**Figure 1:**
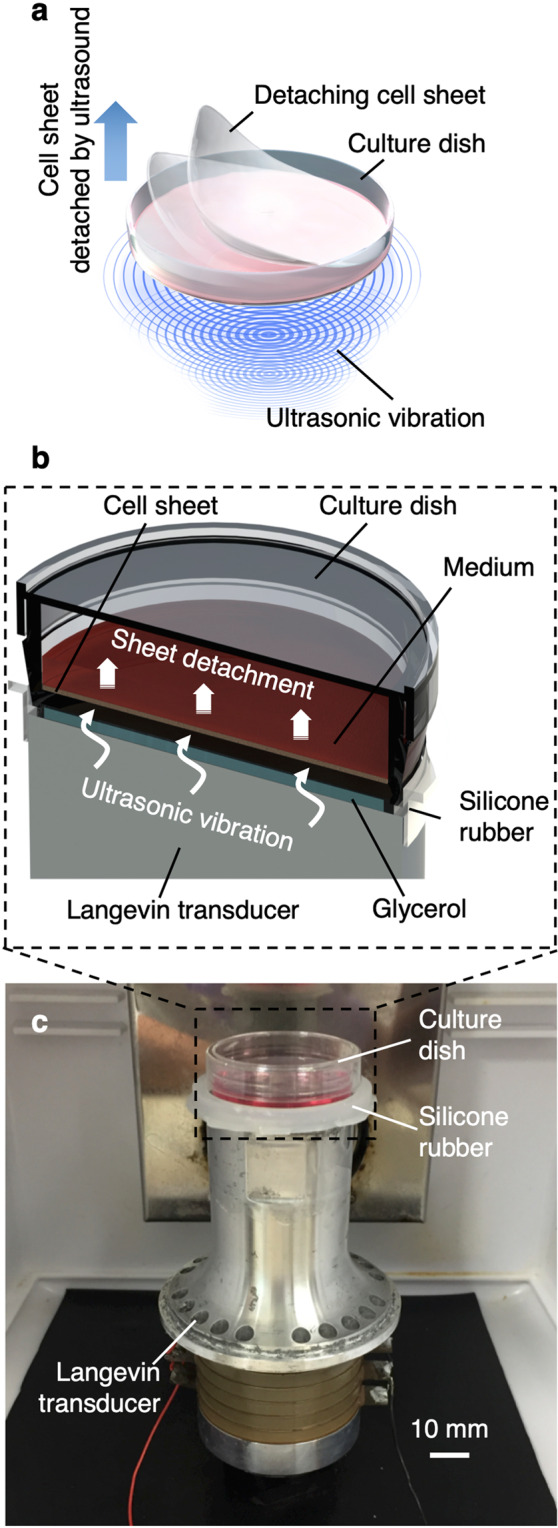
Cell sheet-detaching process using ultrasonic vibration. (**a**) Conceptual image of cell sheet detachment. (**b**) Ultrasonic vibration is applied to cells to detach them from the bottom of the dish. (**c**) Cell sheet-detaching system in an incubator.

**Figure 2:**
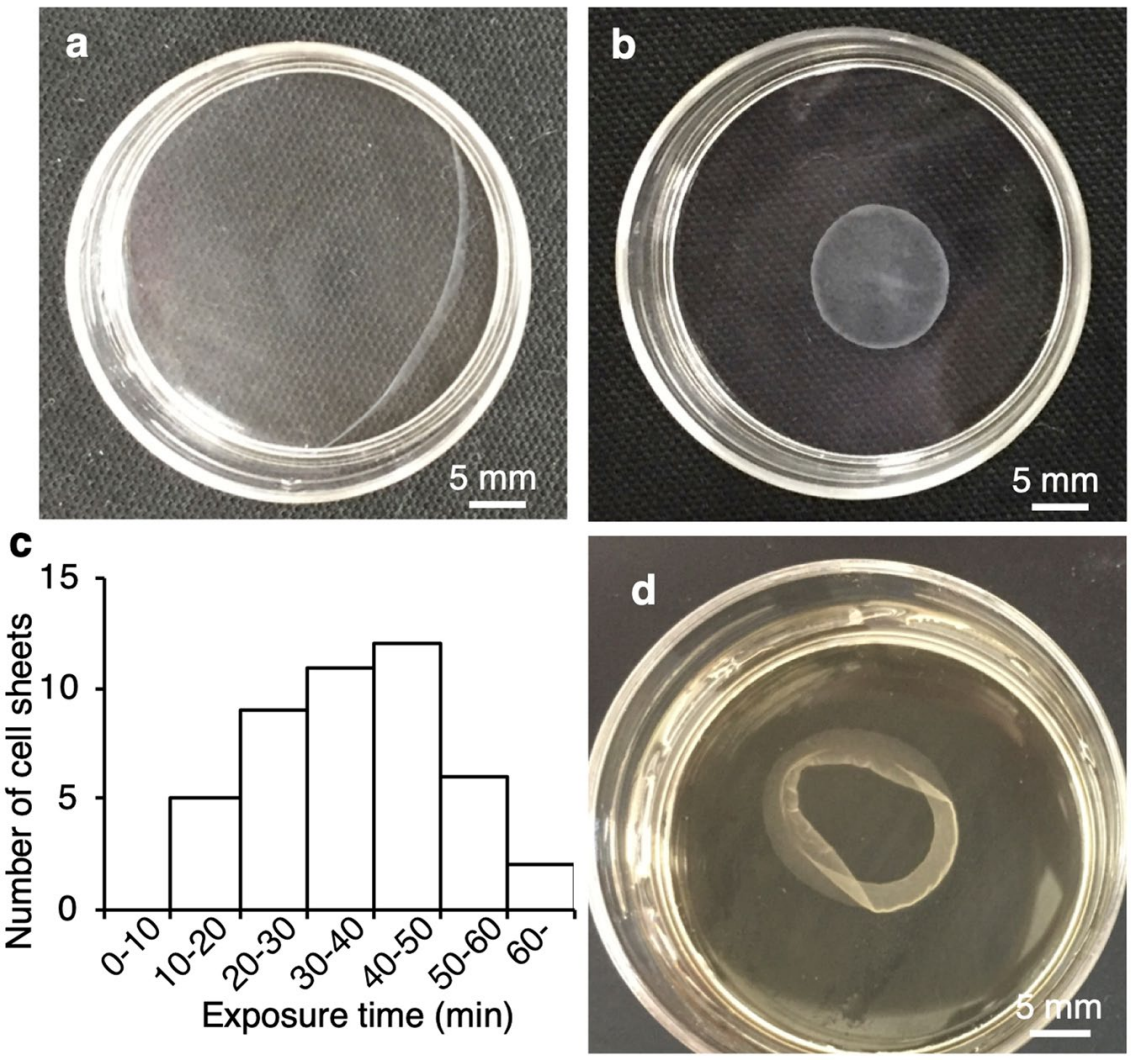
Cell sheet detachment by ultrasonic vibration. Cell sheets after ultrasonic exposure for 1 h at input voltages of (**a**) 12.5 V or (**b**) 25 V. (**c**) Number of cell sheets successfully detached with the corresponding duration of ultrasonic exposure and input voltage of 25 V. The total number of trials was 45. “Number of cell sheets” indicates how many cell sheets were detached at each 10 min of ultrasonic exposure. (**d**) Appearance of cell sheets detached at an input voltage of 25 V.

We mainly used a 35-mm culture dish to demonstrate our concept as shown in Fig. 1a, although we showed cell sheet detachment from culture dishes and flasks in our study. Mouse myoblasts (C2C12 cell line) were seeded, cultured for 3 days to reach confluency, and exposed to ultrasonic vibration from underneath in an incubator as shown in Fig. 1. The ultrasonic vibration was generated by a Langevin transducer with input voltages of 12.5 or 25 V. Figure 2a, b show cell sheets after ultrasonic exposure for 1 h at 12.5 and 25 V, respectively. The cell sheet was successfully detached by ultrasonic vibration excited at 25 V, but not completely detached at 12.5 V within 1 h of exposure to ultrasonic vibration. As shown in Fig. 2b, the cell sheet had shrunk after detaching from the dish, which is also typical in the conventional method^26,27^. To detach cell sheets, 1 h is the standard time required in the conventional method^9^. Figure 2c shows the number of successfully detached cell sheets (vertical axis) with the corresponding duration of ultrasonic exposure (horizontal axis) at an input voltage of 25 V. The total number of trials was 45. By exposure to ultrasonic vibration from underneath the dish, a cell sheet could be detached with a probability of 95.6% within 1 h. Furthermore, Fig. 2d shows the appearance of a cell sheet being detached with an input voltage of 25 V, demonstrating that the cell sheet was detached from the circumference.

In addition to the results of cell sheet detachment from a 35-mm culture dish, we detached a cell sheet from a T25 flask (90025, TPP Techno Plastic Products AG, Trasadingen, Switzerland) to demonstrate the general versatility of our method (See Supplementary Note 1).

### Cell viability, ECM, and morphology

**Figure 3:**
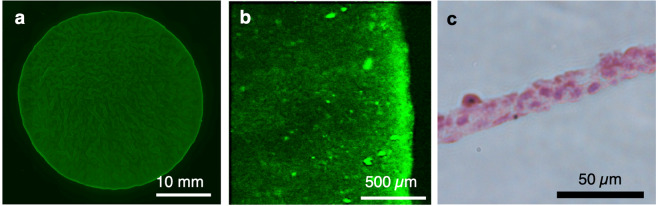
Cell sheet evaluation immediately after detachment. (**a**) Entire calcein-stained cell sheet and (**b**) the edge of a fibronectin-stained cell sheet observed by fluorescence microscopy. (**c**) Cross-section view of a HE-stained cell sheet observed by phase-contrast microscopy.

Live cells in a cell sheet were stained with calcein-AM to estimate cell viability. As shown in Fig. 3a, the cell sheet detached by ultrasonic exposure (25 V, 1 h) was entirely stained with calcein-AM, indicating that the cell sheet consisted of live cells. To evaluate the ECM on the cell sheet, fibronectin, a type of ECM protein, was stained on the cell sheet and in the dish on which the cell sheet had been attached. Figure 3b shows stained fibronectin located only on the cell sheet, indicating that the fibronectin was intact on the cell sheet and did not remain on the dish surface. Therefore, the developed method could fabricate a cell sheet with rich fibronectin similar to the conventional method using a pNIPAAm-coated culture dish^28^. Fibronectin is related to cell functions such as adhesion. To observe morphology of the cell sheet, haematoxylin and eosin (HE) staining was performed as shown in Fig. 3c, and HE staining was performed on a cell sheet detached by the conventional method in Fig. S2. Cells were observed as attached to each other in the cross section of the cell sheet. The thickness of the cell sheet was less than 40 µm. Furthermore, there were no folded parts, indicating that the cell sheet had a homogeneous thickness. In short, there was no morphological difference in either method.

**Figure 4:**
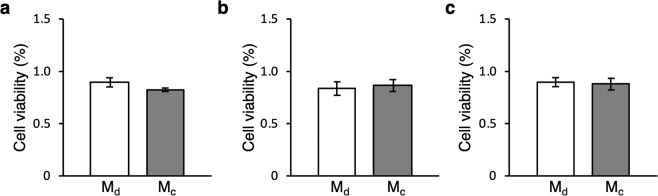
Results of cell sheet viability assays. Evaluation was performed (**a**) immediately before detachment, (**b**) immediately after detachment, and after 24 h of culture (mean ± SD, *n* = 3). Note that Md and Mc represent the proposed and conventional methods, respectively.

We also compared the viabilities of the cell sheets, as shown in Fig. 4, and found no difference using either method at each time point.

### Cell sheet metabolism

**Figure 5:**
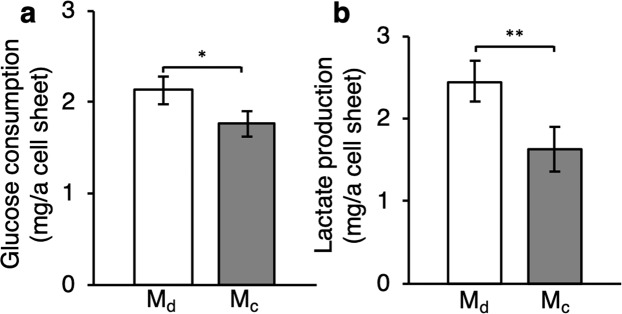
Results of cell sheet metabolism assays. (**a**) Glucose consumption and (**b**) lactate production after 24-h of culture (mean ± SD, *n* = 4, *p < 0.05; **p < 0.01). M_d_ and M_c_ represent the _d_eveloped and conventional methods, respectively. These assays were conducted after culturing cell sheets detached by each method for 24 h. Detached cell sheets were transferred to another culture dish and cultured with 2 mL culture medium. Finally, the supernatant was collected and monitored after 24 h of culture.

For therapy using cell sheets, paracrine effects are important^6,29^. Cells are expected to secrete cytokine after transplantation. Therefore, cell activity should be maintained^30^. Estimation of cell sheet activity was conducted at 24 h of culture after detachment. Metabolic activity of the cell sheet detached by the developed method was evaluated by comparison with a cell sheet detached by the conventional method (control). Except for the culture dish, all culture conditions before detachment were the same as the developed method. Figure 5 shows the comparisons of glucose consumption and lactate production between the cell sheets detached by the developed and conventional methods at 24 h of culture after detachment. Both indexes of the cell sheet fabricated by the developed method were statistically higher than those of the cell sheet fabricated by the conventional method. These results suggest that the developed method generates cell sheets with higher metabolism compared with the conventional method.

### Protein assays of cell sheets

**Figure 6:**
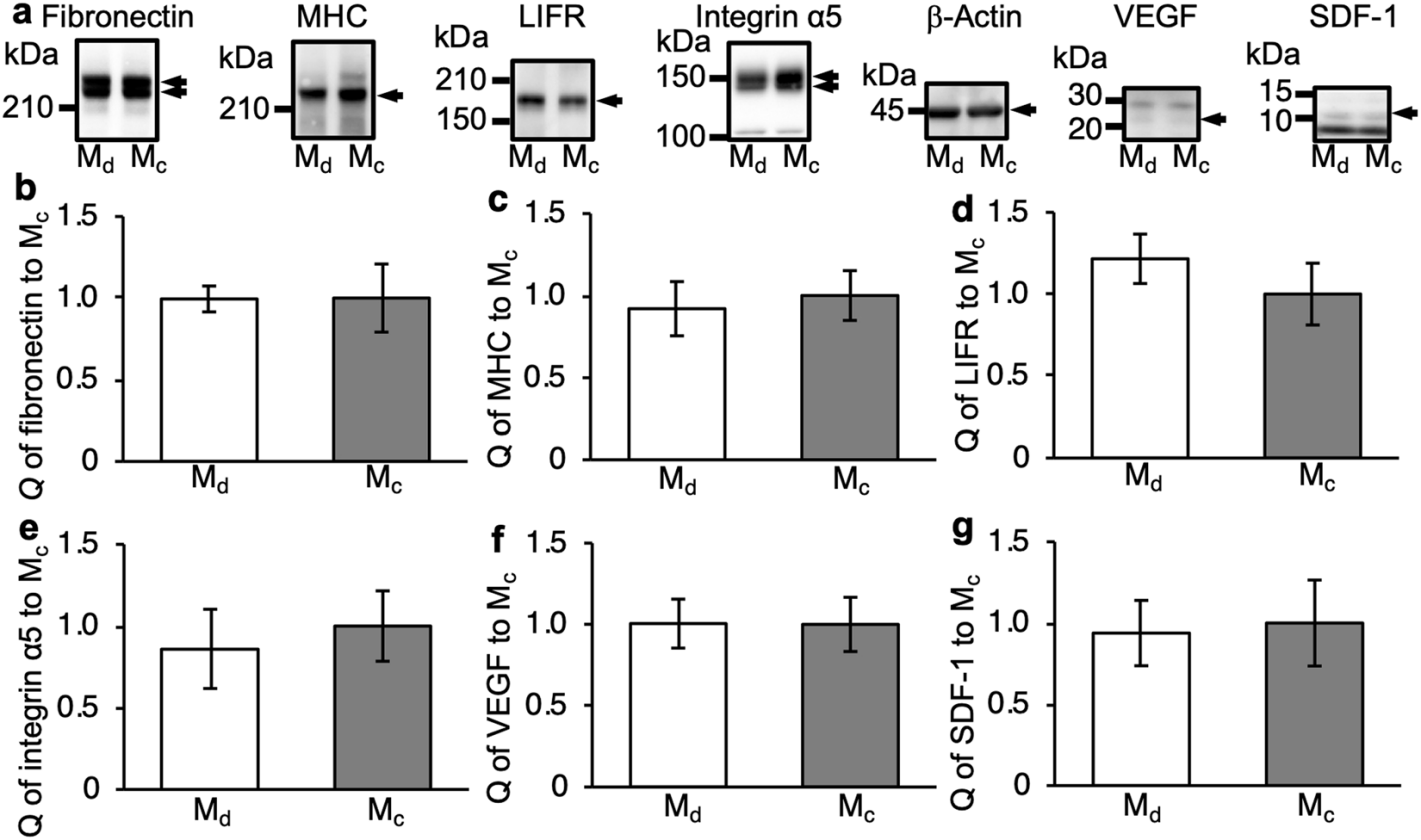
Comparison of protein expression in cell sheets. (**a**) Cell sheets collected by the developed method (M_d_) and conventional method (M_c_) were lysed in SDS-PAGE sample buffer, and proteins were analysed by western blotting with the following antibodies: anti-fibronectin (220 kDa, first panel), anti-myosin heavy chain (MHC, 220 kDa, second panel), anti-leukaemia inhibitory factor receptor (LIFR, 190 kDa, third panel), anti-integrin α5 (150 kDa, fourth panel), anti-β-actin (45 kDa, fifth panel), anti-vascular endothelial growth factor (VEGF, 22 kDa, sixth panel), and anti-SDF-1 (10 kDa, seventh panel). Arrows indicate target bands. Relative protein quantities (Q) of (**b**) fibronectin, (**c**) MHC, (**d**) LIFR, (**e**) integrin α5, (**f**) VEGF, and (**g**) SDF-1 were measured using their band densities on western blots. Protein quantities were normalized to the band density of β-actin and expressed as the quantity relative to the conventional method (mean ± SD, *n* = 4). Western blots were cropped for clarity; uncropped images can be found in Supplementary Fig. S3.

Seven kinds of proteins were quantified by western blotting in cell sheets fabricated by the developed and conventional methods, including myosin heavy chain (MHC), leukaemia inhibitory factor receptor (LIFR), integrin α5, fibronectin, stromal cell-derived factor 1 (SDF-1), vascular endothelial growth factor (VEGF), and β-actin. MHC, LIFR, integrin α5, and β-actin are cellular proteins, whereas fibronectin is an ECM protein, and SDF-1 and VEGF are cytokines^21,29,31^. MHC and β-actin are located in the cytoplasm, while LIFR and integrin α5 are located on the cell membrane. MHC and LIFR were quantified as differentiation indexes. Integrin α5 and fibronectin are related to cell adhesion. SDF-1 recruits several kinds of stem/progenitor cells, and VEGF induces angiogenesis^32^. After transferring the cell sheets to 35-mm culture dishes, they were cultured for 24 h in 2 mL growth medium, and then protein assays were conducted. The results of western blotting are shown in Fig. 6. The band density was normalized to the β-actin band and expressed as the relative quantity to the data obtained from cells detached by the conventional method. The results indicated that expression of the abovementioned proteins was not altered by the developed method.

### mRNA expression assays of cell sheets

**Figure 7:**
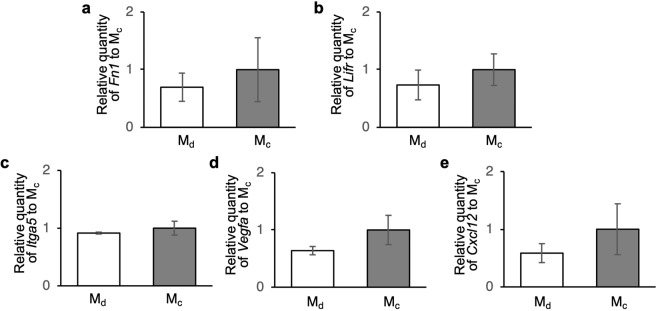
Comparison of mRNA expression in cell sheets. Relative mRNA expression of (**a**) *Fn1*, (**b**) *Lifr*, (**c**) *Itga5*, (**d**) *Vegfa*, and (**e**) *Cxcl12* in cell sheets was quantified by RT-qPCR. Cell sheets were collected by the developed and conventional methods. mRNA quantities were normalized to β-actin mRNA and then calibrated by the relative quantity to the quantity obtained from cells detached by the conventional method (mean ± SD, *n* = 3). The relative mRNA quantities of *Myh2* could not be calculated the using 2^−ΔΔCt^ method, in which C_t_ is the threshold cycle, because its mRNA expression was not detected in some RT-qPCRs.

Expression of seven mRNAs was quantified by reverse transcription-quantitative polymerase chain reaction (RT-qPCR) in cell sheets fabricated by the developed and conventional methods, including myosin heavy polypeptide 2 (*Myh2*), leukaemia inhibitory factor receptor α (*Lifr*), integrin α5 (*Itga5*), fibronectin 1 (*Fn1*), C-X-C motif chemokine ligand 12 (*Cxcl12*), vascular endothelial growth factor A (*Vegfa*), and β-actin (*Actb*). Each gene encoded the following proteins: MHC, LIFR, integrin α5, fibronectin, SDF-1, VEGF, and β-actin, respectively. Cell sheets were cultured for 24 h in 2 mL growth medium after detachment and then RT-qPCR was conducted. The results of RT-qPCR are shown in Fig. 7. The mRNA quantities were normalized to β-actin mRNA and then calibrated by the relative quantity to the quantity obtained from cells detached by the conventional method. As a result, there was no significant difference in mRNA expression of *Lifr*, *Itga5*, *Fn1*, *Cxcl12*, and *Vegfa* between the samples collected by the developed or conventional methods. The relative mRNA quantities of *Myh2* could not be calculated by the 2^−ΔΔCt^ method, in which C_t_ is the threshold cycle, because its mRNA expression was not detected in some RT-qPCRs. These results showed that the mRNA expression in cell sheets was not altered by the proposed method.

## Discussion

We demonstrated that a cell sheet of mouse myoblasts could be detached from clinically ubiquitous cell culture by suitable ultrasonic exposure, which are commonly used in proof-of-concept tissue engineering and cell sheet studies^9^. Furthermore, the metabolism of the cell sheet fabricated by the developed method was statically improved compared with the conventional method, while cell viability, protein expression, and mRNA expression showed no significant differences. Therefore, we have provided a simple method to detach a cell sheet from a clinically ubiquitous cell culture vessel supply without using any additional materials or chemicals. In addition, the fabricated cell sheet had a higher metabolism compared with a cell sheet prepared by the widely used conventional method. This critical improvement relied on replacement of the temperature-responsive polymer by ultrasonic exposure, because other experimental conditions, such as the number of seeded cells, growth medium, and culture time, were the same in both the developed and conventional methods.

As shown in Figs. 3 and 4, cell viability, the fibronectin location, and morphology were evaluated immediately after detachment of the cell sheet. The cell sheet consisted of living cells, had rich fibronectin, and a homogeneous three-dimensional morphology with a maximum thickness of less than 40 µm. Cell viability is undoubtably an important index for cell activity, and the ultrasonic exposure under the conditions of the developed method did not have any negative effects on viability. Although there is the possibility of cavitation with ultrasonic exposure, data shown in Fig. 3a eliminated this possibility, which is always a concern in a system using kHz range ultrasonic vibration^33^. Once cavitation occurs, bubbles should induce a strong shockwave that can crush cells when they collapse^33^. Accordingly, a cell sheet is likely to break. However, Fig. 3a shows that the cell sheet did not break and consisted of live cells, demonstrating that cavitation did not occur or at least did not damage cell sheets in our method. The location of fibronectin shown in Fig. 3b also indicates a positive aspect of the developed method. Fibronectin, which is a type of ECM protein stained in this study, is related to cell activity, especially adhesion^33^. Fibronectin was intact on the cell sheet and not on the dish, suggesting that the fabricated cell sheet had high activity at least from the viewpoint of initial adhesion, which is important for engraftment upon transplantation^34^. The homogeneous cell sheet morphology with the maximum thickness of 40 µm revealed by HE staining (Fig. 3c) minimizes the possibilities of partial differentiation and necrosis. The cell environment affects their differentiation. Therefore, a homogeneous thickness is required to fabricate cell sheets with homogeneous functions. Furthermore, necrosis occurs in a tissue with a thickness of 150–200 µm or thicker^35^. Thus, as indicated by the results of estimations immediately after detaching a cell sheet by ultrasonic exposure, the developed method did not exert any negative effects on the fabricated cell sheet.

As an estimation of cell activity after transplantation, Fig. 5 shows the cell metabolism represented by glucose consumption and lactate production that are generally used as cell metabolism indexes^33^. Cell sheets detached by the developed method had statistically higher metabolism than those detached by the conventional method. In the conventional method, temperature is lowered to 20 °C for 1 h, whereas temperature is not reduced in the developed method. It is known that temperature reduction may decrease cell metabolism^36^. Thus, we believe that the main reason for the improvement in cell metabolism by the developed method is the absence of temperature reduction. Because cell metabolism is related to cell activity including secreting cytokines and cell adhesion^37,38^, the developed method contributes to producing a highly active cell sheet.

Protein and mRNA expression is important to evaluate the normality of cells. The results of protein quantifications are shown in Figs. 6 and 7. For convenience, we made discussion basing protein assay. Previous studies have reported that vibration affects the differentiation rate of myoblasts^39^. However, the expression of MHC used as a differentiation index was not altered in cell sheets detached by the developed method. Furthermore, expression of LIFR, which is related to several cell functions including differentiation, was not altered^40^. Thus, the ultrasonic exposure in the developed method had no effect on the differentiation rate of myoblasts. Integrin α5 and fibronectin were quantified to evaluate the cell adhesion ability. Integrin α5 is mainly related to cell-fibronectin adhesion, and fibronectin is an ECM protein that acts an anchor for cell adhesion. Because the results showed that both integrin α5 and fibronectin in the cell sheet detached by the developed method were intact, we concluded that the detached cell sheet had a good adhesion ability. Integrin α5 and fibronectin are also related to other cell activities including proliferation, differentiation, migration, and secreting ECM proteins and cytokines^41–43^. Therefore, these functions of the cells would be conserved when the cell sheet is detached by the developed method. Cytokines such as SDF-1 and VEGF, which were also quantified, are not directly related to cell sheet functions. However, they play very important roles in clinical applications. In regenerative medicine, cell sheets are transplanted onto organs. For example, myoblast cell sheets are transplanted onto a heart to improve cardiac functions^44^. The efficiency of the therapy depends on the cytokine-secreting ability of the cells. Because SDF-1 recruits several kinds of stem/progenitor cells, it is a key protein in regenerative medicine^45,46^. Furthermore, VEGF induces strong angiogenesis that is important to maintain the transplanted cell sheet, because tissues cannot survive without a proper blood supply^32^. Cytokine expression in a cell sheet detached by the developed method was not altered. Additionally, the proteins quantified in this study were located in the cytoplasm, membrane, ECM, and supernatant. Consequently, we can conclude that, regardless of the protein location, the ultrasonic exposure did not affect protein expression. Furthermore, mRNA analyses demonstrated that our detached cell sheets were intact. These results were expected because the cell sheet should be detached forcibly and not by chemical reactions^22^. Cell viability was also unaffected by the developed method. Thus, we concluded that the developed method forcibly, but gently, detached a cell sheet by acoustic radiation force.

The developed and conventional methods can be compared from several viewpoints. From the viewpoint of cost, although our method requires an ultrasound transducer, costly expendable supplies are not required. The device in this study has a driving frequency similar to a ubiquitous device such as an ultrasonic cleaner. Thus, it is very realistic for bioengineering laboratories to have access to the developed method. The required time for cell sheet detachment was similar to that for the conventional method, but a cool incubator is not required. The functions of the cell sheets detached by either method were similar except for the improved metabolism with our method. In conclusion, the cell sheet fabrication method developed in this study can be another novel option for cell sheet detachment in addition to the conventional cell sheet fabrication method. Because a cell sheet can be fabricated in clinically ubiquitous cell culture vessel, the developed method has great potential as a standard method for cell sheet fabrication with dramatically reduced cost. Furthermore, as shown in Supplementary Note 1, cell sheets can be detached from not only a 35-mm culture dish but also a T25 flask by our method. This feature indicates that the concept of our method can be applied to any culture vessel, which enables fabrication of large cell sheets consisting of vastly living cells. In fact, our method was demonstrated by one murine cell line, which might be a limitation of our study. However, testing of more physiologically relevant models will make clinical translation possible. We believe the above findings contribute to the development of tissue engineering and regenerative medicine.

## Methods

### Cell sheet-detaching system and conditions

The cell sheet-detaching system is shown in Fig. 1b. A 35-mm culture dish (430165; Corning Inc., Corning, NY, USA) was placed on a Langevin transducer (HEC-5020P6BHF JW; Honda Electronics Co., Ltd., Aichi, Japan) with 450 µL glycerol between them. Because acoustic impedance of glycerol was almost equivalent to that of the culture dish, 93.5% of the acoustic intensity was propagated to the cells (see Supplementary Note 3). To hold the glycerol and culture dish appropriately, a dish holder made of silicone rubber with an inner diameter of 37 mm was placed on the transducer. The cell sheet-detaching system was placed in a 5% CO_2_ humidified atmosphere incubator (AS-203M; AS ONE, Osaka, Japan) at 37 °C during experiments. As a result, a cell sheet was detached from the culture surface by the ultrasonic vibration generated by the longitudinal resonance of the transducer (see Supplemental Note 4). Although the transducer resonated at 19.68 kHz, the resonance frequency may slightly alter because of contact with the glycerol and dish containing culture medium. Therefore, we alternated the driving frequency between 19.6 kHz and 19.8 kHz with a sweep cycle of 100 ms to ensure excitation of resonance vibration of the transducer during the cell sheet-detaching experiments. Input voltage to the transducer was set at 12.5 or 25 V to prevent a possible temperature rise due to ultrasonic exposure (see Supplementary Note 5). We exposed cells to the ultrasonic vibration for up to 1 h. The input signal to the transducer was generated by a function generator (WF1946B; NF Corporation, Kanagawa, Japan) and amplifier (HSA4051; NF Corporation, Kanagawa, Japan).

### Cell preparation

Mouse myoblasts (C2C12) (RCB0987; Riken Bio Resource Center, Ibaraki, Japan) were used in experiments. Cells were cultured in growth medium in a 5% CO_2_ humidified atmosphere incubator at 37 °C. Cell passaging was performed by trypsinization in 0.05% trypsin-EDTA (25300; Life Technologies, Carlsbad, CA, USA). For cell sheet detachment, 6 × 10^5^ cells were seeded into a clinically ubiquitous 35-mm culture dish (430165; Corning Inc.) or UpCell^®^ (CS3007; CellSeed, Tokyo, Japan) and cultured for 3 days in 2 mL growth medium [Dulbecco’s modified Eagle’s medium/F12 supplemented with 10% fetal bovine serum (2917254; MP Biomedicals, Santa Ana, CA, USA)]. For the conventional detaching method, the UpCell^®^ was placed in the 5% CO_2_ humidified atmosphere incubator at 20 °C.

### Cell staining

Immediately after detaching the cell sheet, immunofluorescence staining was conducted to evaluate viability of the cell sheet and localization of fibronectin. To assess viability, live cells were stained with calcein-AM (C0875; Sigma-Aldrich, St. Louis, MO, USA), and fibronectin was stained with a mouse anti-human fibronectin monoclonal antibody (ab194395; Abcam, Cambridge, UK) and Alexa Fluor 488-conjugated goat anti-mouse IgG H&L (ab150117; Abcam). Before calcein treatment, the medium was removed from the dish, and the cell sheet was washed twice with phosphate-buffered saline (PBS) (T900; Takara Bio Inc., Shiga, Japan). After washing, the cell sheet was treated with a 500-fold dilution of calcein-AM in serum-free culture medium (D-MEM/F12) for 30 min. Fibronectin was stained by the following procedure. The detached cell sheet and any ECM remaining on the culture dish surface were fixed with cold methanol (25183-00; Kanto Chemical Co., Inc., Tokyo, Japan). Cells were permeabilized with 0.1% Triton X-100 (C0875; Sigma-Aldrich) for 15 min. After blocking non-specific binding sites with 10% goat serum (005-000-121; Jackson ImmunoResearch, West Grove, PA, USA) in PBS for 1 h at room temperature, cells in the culture dish were incubated with 1 µL/1 mL anti-fibronectin antibody. Then, they were incubated with a 1,000-fold dilution of Alexa Fluor 488-conjugated goat anti-mouse IgG H&L (ab150117; Abcam) for 30 min.

HE staining was conducted to confirm the three-dimensional structure of the cell sheet. After detachment, a cell sheet was fixed with a 4% paraformaldehyde solution (09154-56; Nacalai Tesque, Kyoto, Japan) overnight at 4 °C and then immersed in 20% sucrose (193-00025; Wako Pure Chemical Corporation, Osaka, Japan) in PBS for 7 h at 4 °C. The cell sheet was fixed with an artificial support membrane, Cell Shifter™ (CSD001; CellSeed, Tokyo, Japan) for cryosectioning. After these treatments, the specimen was frozen in Tissue-Tek^®^ OCT compound (4583; Sakura Finetek Japan, Tokyo, Japan) and cryosectioned at a thickness of 6 µm. The cross-section was stained with HE by a conventional method^35^. Finally, images were captured under an inverted microscope (ECLIPSE Ti; Nikon Corporation, Tokyo, Japan).

Staining with a Trypan blue solution (15250061; Thermo Fisher Scientific, Waltham, MA, USA) at each time point was performed to evaluate cell viabilities.

### Glucose consumption and lactate production

Metabolism of a cell sheet was evaluated by measuring glucose consumption and lactate production in the culture supernatant after 24 h of culturing a cell sheet in a 35-mm clinically ubiquitous cell culture dish with 2 mL growth medium. Cell sheets were transferred to another dish after detachment from the original dish. The concentrations of glucose and lactate were determined by a glucose assay kit (GAHK-20; Sigma-Aldrich) and lactate assay kit (K607-100, BioVision, Milpitas, CA, USA), respectively^47,48^.

### Western blot analysis

Cell sheets cultured for 24 h after detachment by the developed and conventional methods were lysed in sodium dodecyl sulfate-polyacrylamide gel electrophoresis (SDS-PAGE) sample buffer [1 mL/cell sheet; 50 mM Tris-HCl (pH 6.8), 75 mM dithiothreitol, 2% SDS, 10% glycerol, and 0.001% bromophenol blue] and heated at 70 °C for 5 min. Proteins were resolved by SDS-PAGE (7.5% Tris-HCl gel) and transferred onto polyvinylidene fluoride membranes. The membranes were treated with Blocking One (Nacalai Tesque) for 30 min with shaking to block non-specific binding of antibodies to the membranes. Then, the membranes were treated with the following antibodies in 5% Blocking One/Tris-buffered saline (TBS; pH 7.4) for 12 h with shaking: 0.4 µg/mL mouse anti-human fibronectin monoclonal antibody (ab194395; Abcam), 2 µg/mL mouse anti-chicken MHC monoclonal antibody (MF20; R&D systems, Minneapolis, MN, USA), 0.4 µg/mL rabbit anti-human LIFR polyclonal antibody (22779-1-AP; Proteintech, Rosemont, IL, USA), 500-fold dilution of rabbit anti-human integrin α5 polyclonal antibody (#98204; Cell Signaling Technology, Boston, MA, USA) 1,000-fold dilution of rabbit anti-mouse β-actin polyclonal antibody (#4967; Cell Signaling Technology), 1 µg/mL rabbit anti-human SDF-1 polyclonal antibody (41422; Signalway Antibody, College Park, MD, USA), and 0.5 µg/mL rabbit anti-human VEGF polyclonal antibody (R30265; NSJ Bioreagents, Carmel Mountain Ranch, CA, USA). After washing three times with TBS containing 0.05% Tween 20 (TBST), the membranes were treated with a 10,000-fold dilution of horseradish peroxidase (HRP)-conjugated goat anti-mouse IgG (12–349; Sigma-Aldrich) or anti-rabbit IgG (62–6120; Thermo Scientific, Waltham, MA, USA) for 30 min with shaking. After washing three times with TBST, protein bands were visualized with Immobilon Western chemiluminescent HRP substrate (Millipore, Billerica, MA, USA), scanned, and analysed using a LAS-4000 image analyser (Fujifilm, Tokyo, Japan). The band density was normalized to the β-actin band and expressed as the quantity relative to the conventional method.

### RT-qPCR analysis

Relative mRNA expression was measured by RT-qPCR using total RNA extracted from a cell sheet cultured for 24 h after detachment by each method. Total RNA was extracted using a NucleoSpin RNA (740955.50; Takara Bio Inc.) and quantified using a Thermal Cycler Dice Real Time System Lite (TP700; Takara Bio Inc.). RNA was reverse transcribed into cDNA with PrimeScript Master Mix (Perfect Real Time) (RR036A; Takara Bio Inc.), an oligo (dT) primer, and random hexamer primers for 15 min at 37 °C and then 5 sec at 85 °C. The cDNA concentration was quantified using a Biophotometer (6131; Eppendorf, Hamburg, Germany) and then decreased with RNase-free water (9012; Takara Bio Inc.) to 10 ng/µL cDNA. RT-qPCR was conducted in the Thermal Cycler Dice Real Time System Lite for 30 sec at 95 °C and then 60 cycles of 5 sec at 95 °C and 30 sec at 60 °C. RT-qPCRs contained 12.5 µl TB Green Premix Ex Taq II (Tli RNaseH Plus) (RR820A; Takara Bio Inc.), 20 ng cDNA, 0.4 µM of each forward and reverse primer, and 8.5 µL RNase-free water. The primer sequences are shown in Table 1. RT-qPCR was performed in technical triplicates for each primer pair and cDNA sample. In addition, the reactions were conducted as biological triplicates under similar conditions. To verify that primer dimers were not responsible for the obtained fluorescence signals, melting curve analysis of amplicons was performed for each primer pair. Negative control reactions without the templates were also performed to ensure the data quality. Relative mRNA expression was normalized to β-actin mRNA and then calibrated by the relative quantity to the quantity obtained from cells detached by the conventional method. The fold change was calculated using the 2^−ΔΔCt^ method in which C_t_ is the threshold cycle.

**Table 1:** RT-qPCR primer sequences in this study.

Gene name	Gene bank number	Sequence(5′–3′)	Tm (°C)	Product size (bp)
*Actb*	NM_007393.5	Forward CACCGTAAAGACCTCTATGCCAAC	64.2	171
		Reverse ATGGAGCCACCGATCCACA	65	
*Myh2*	NM_001039545.2	Forward ATTCTCAGGCTTCAGGATTTGGTG	64.9	114
		Reverse CTTGCGGAACTTGGATAGATTTGTG	65	
*Lifr*	NM_001113386.1	Forward TCAAACAGCACGGAGACTGTCATA	64.2	88
		Reverse CCTGGTTAGTGCACCCATAGAGGTA	64.6	
*Fn1*	NM_001276408.1	Forward GCTTTGGCAGTGGTCATTTCAG	64.2	134
		Reverse ATTCCCGAGGCATGTGCAG	64.6	
*Itga5*	NM_010577.4	Forward GAAGCTCTGAAGATGCCCTACCA	64.1	124
		Reverse TGATGATCCACAACGGGACAC	64.2	
*Cxcl12*	NM_013655.4	Forward CAGAGCCAACGTCAAGCATC	62	110
		Reverse TTAATTTCGGGTCAATGCACAC	62	
*Vegfa*	NM_001025250.3	Forward ACATTGGCTCACTTCCAGAAACAC	63.8	108
		Reverse TGGTTGGAACCGGCATCTTTA	64.7	

### Statistical analysis

The statistical significance of differences was evaluated by Welch’s t-test. *P* < 0.05 was accepted as statistically significant.

## Supplementary information

Supplementary information.

## Data Availability

Data supporting the findings of this study are available in the article and Supplementary information files, or from the corresponding author upon request. All data generated and analysed during this study are included in this published article (and its Supplementary Information file).
