# Supplementary material for: Detachment of cell sheets from clinically ubiquitous cell culture vessels by ultrasonic vibration
Source: Sci Rep. 2020 Jun 11;10:9468. doi: 10.1038/s41598-020-66375-1 (PMC7289836; doi:10.1038/s41598-020-66375-1)
Supplement: Supplementary file 1 — Supplementary information. [file 41598_2020_66375_MOESM1_ESM.pdf]

Detachment of cell sheets from clinically ubiquitous cell culture vessels by ultrasonic vibration

Chikahiro Imashiro[1, 2], Makoto Hirano[3], Takashi Morikura[4], Yuki Fukuma[4], Kiyoshi Ohnuma[5,6], Yuta Kurashina[7,1] Shogo Miyata[1]& Kenjiro Takemura[1]*

[1]Department of Mechanical Engineering, Keio University, 3-14-1 Hiyoshi, Kohoku-ku, Yokohama 223-8522, Japan. [2]Institute of Advanced Biomedical Engineering and Science, Tokyo Women's Medical University, TWIns, 8-1 Kawada-cho, Shinjuku-ku, Tokyo, Japan. [3]Department of Pharmacy, Yasuda Women's University, 6-13-1 Yasuhigashi, Asaminami-ku, Hiroshima, Japan. [4] School of Science for Open and Environmental Systems, Graduate School of Science and Technology, Keio University, 3-14-1 Hiyoshi, Kohoku-ku, Yokohama 223-8522, Japan. [5]Department of Bioengineering, Nagaoka University of Technology, 1603-1 Kamitomioka, Nagaoka, Niigata 940-2188, Japan. [6]Department of Science of Technology Innovation, Nagaoka University of Technology, 1603-1 Kamitomioka-cho, Nagaoka, Niigata 940-2188, Japan. [7]Department of Materials Science and Engineering, School of Materials and Chemical Technology, Tokyo Institute of Technology, Yokohama 226-8503, Japan.

Correspondence and requests for materials should be addressed to K. T. (email: takemura@mech.keio.ac.jp).

Supplementary Note 1

In addition to cell detachment from a 35-mm culture dish, we detached a cell sheet from a T25 flask (90025, TPP Techno Plastic Products AG, Trasadingen, Switzerland) to demonstrate the general versatility of our method. Figure S1 shows cell sheet detachment from a T25 flask. A total of 1×10^7 mouse myoblasts (C2C12 cell line) were seeded into a T25 flask with 5 mL medium and cultured for 1 day to reach confluency. Then, the flask was placed on a Langevin transducer (HEC-45402, HONDA ELECTRONICS CO., LTD., Tokyo, Japan) with a closed cap, and the cells were exposed to ultrasonic vibration from underneath at room temperature. The ultrasonic vibration was generated by the Langevin transducer with a driving frequency sweep between 158.20 and 158.40 kHz with a sweep cycle of 100 ms and input voltage of 87.5 V, which was propagated to the cells via water located between the flask and transducer. Note that the resonance frequency of the transducer was 158.28 kHz. Figure S1a shows the cell sheet being detached from the edge of the flask, and Fig. S1b shows the detached cell sheet. To observe the shape of the detached cell sheet, we transferred it to a 60-mm culture dish (3010-060-MYP, AGC Techno Glass Co., Ltd., Shizuoka, Japan) (Fig. S1c). Furthermore, to estimate viability of the cell sheet, it was stained with calcein-AM (Fig. S1d). As shown in Fig. S1, cell sheets could be detached from T25 flasks, indicating that our method can detach cell sheets from wide range of clinically ubiquitous cell culture vessels.

Supplementary Note 2

To compare the morphology of the cell sheet detached with our and conventional method, a HE stained image of cell sheet detached with conventional method was shown in Supplementary Fig. S2.

Supplementary Note 3

To propagate vibration from one medium to another, the difference in acoustic impedance of the medium determines the propagation efficiency. The propagation coefficient (*T*) can be expressed as

$$T = \frac{2Z_A}{Z_A + Z_B} \quad (1)$$

where Z_A and Z_B represent the acoustic impedances of the two media. Acoustic impedances of glycerol, polystyrene, which comprised the culture dish, and water, which mainly comprised the culture medium, are shown in Supplementary Table 1[S1]. Because ultrasonic vibration from the ultrasonic transducer propagated through glycerol, a dish, and culture medium, the vibration amplitude propagated to the culture medium was 123.9% of that at the transducer surface when

attenuation is ignored. A reason for such disregard is the short thickness of the glycerol layer compared with the wavelength of the ultrasound (97–98 mm). Furthermore, acoustic intensity, I, can be expressed as

$$I = 2\rho c\pi^2 f^2 A^2 \qquad (2)$$

where ρ, c, f, and A are the medium density, sound speed in the medium, vibration frequency, and vibration amplitude, respectively. Sound speed and the density of each material are shown in Supplementary Table 1. Based on eq. (2) and the physical properties listed in Supplementary Table 1, 93.5% of the acoustic intensity generated at the ultrasonic transducer was transferred to the cells.

Supplementary Table 1 Physical properties of each material

	Glycerol	Polystyrene	Water
Acoustic impedance (N・s/m^3)	2.43×10^6	2.49×10^6	1.48×10^6
Sound of speed (m/s)	1923	2350	1483
Density (kg/m^3)	1.263×10^3	1.03×10^3	0.998×10^3

Supplementary Note 4

Vibration characteristics of the Langevin transducer were evaluated. Fig. S4a denotes the relationship between the input frequency and consumption current measured by a current probe (CT2 Current Prove, Tektronix, Inc., OR, USA), Furthermore, Figure S4b shows the vibration amplitude distribution on the transducer surface measured by a laser Doppler vibrometer (LV-1800, ONO SOKKI, Kanagawa, Japan). Note that a dish was placed on the transducer as it was during the cell sheet-detaching experiment. As shown in Fig. S4, the transducer resonated at 19.68 kHz with a uniform distribution of the amplitude (longitudinal mode).

Supplementary Note 5

Three input voltages, 12.5, 25.0, and 37.5 V, were used to evaluate temperature variation in 2 mL of medium with an input frequency sweep between 19.6 and 19.8 kHz. Figure S5 shows the time course of temperature changes over 1 h by measurement using a temperature sensor (TR-71wf; T&D Corporation, Nagano, Japan). Whereas the temperature of the medium reached 42°C or higher at an input voltage of 37.5 V, it remained below 38°C at input voltages of 12.5 and 25.0 V. A previous study has reported that the viabilities of some kinds of cells are affected at 42 ± 0.5°C for 2 h[S2],

whereas ≤38°C is an appropriate temperature for cell culture[S3]. Thus, we set the input voltage at 12.5 or 25.0 V in cell sheet-detaching experiments.

References

S1. Imashiro, C., Kurashina, Y., Kuribara, T., Hirano, M., Totani, K., & Takemura, K., Cell patterning method on a clinically ubiquitous culture dish using acoustic pressure generated from resonance vibration of a disk-shaped ultrasonic transducer. *IEEE Trans. Bio-Med. Eng.* DOI: 10.1109/TBME.2018.2835834 (2018).

S2. Mitri, E. *et al.* Time-Resolved FT-IR Microspectroscopy of Protein Aggregation Induced by Heat-Shock in Live Cells. *Anal. Chem* **87,** 3670–3677 (2015).

S3. Wiklund, M. Acoustofluidics 12: Biocompatibility and cell viability in microfluidic acoustic resonators. Lab Chip. **12**, 2018-2028 (2012).

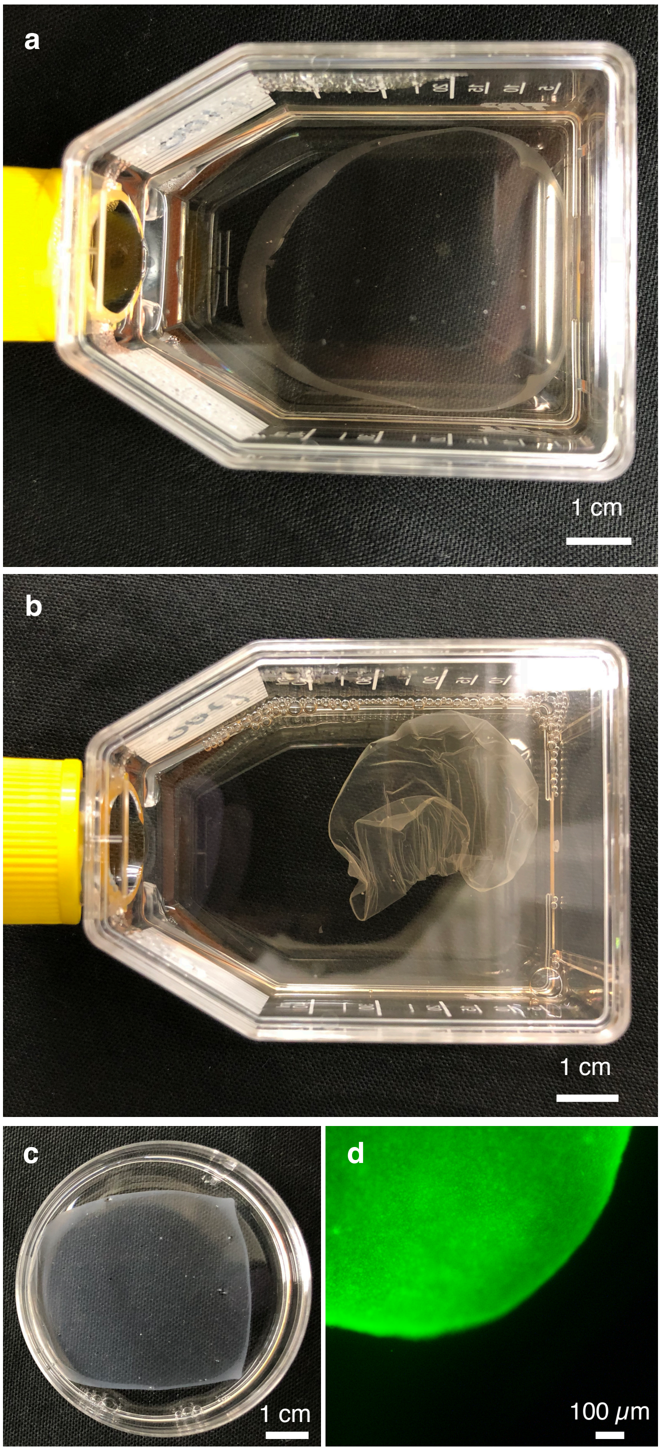

Figure S1. Cell sheet detachment from a T25 flask. (a) Appearance of the cell sheet being detached. **(b)** Cell sheet detached from the flask. **(c)** The detached cell sheet was transferred to a 60-mm dish to observe its morphology. **(d)** A corner of the cell sheet stained with calcein-AM to evaluate viability of the cell sheet. The staining indicated that the cell sheet consisted of live cells.

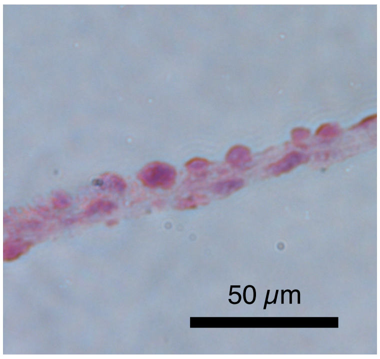

Figure S2. Cross-section view of a HE-stained cell sheet detached by the conventional method.

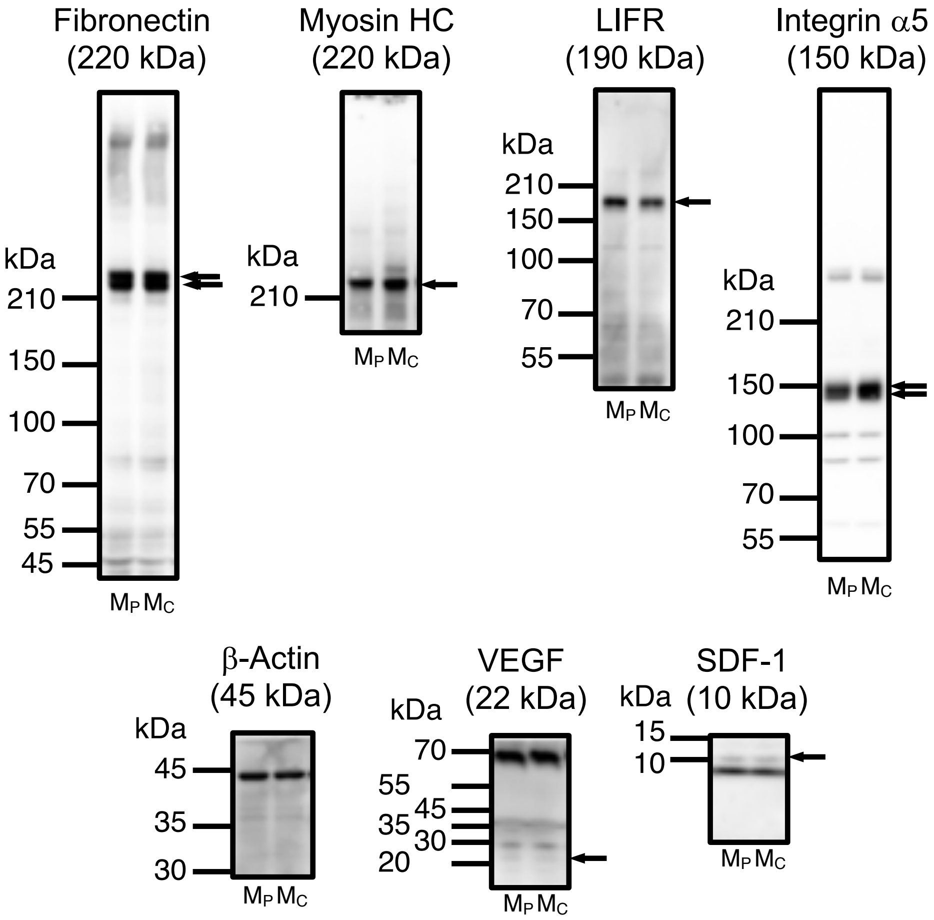

Figure S3. Uncropped western blot gels shown in main manuscript Fig. 6.

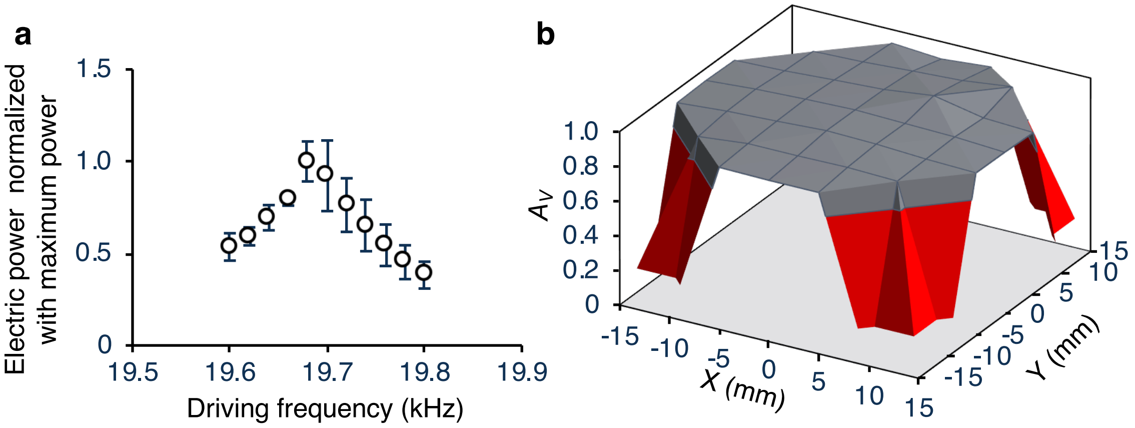

Figure S4. Vibration characteristics of the Langevin transducer. (**a**) Relationship between input frequency and electric power consumption. Electric power was normalized to the maximum value (12.5 W) at resonance. **(b)** Vibration amplitude distribution on the transducer surface measured by a laser Doppler vibrometer. A_v: vibration amplitude normalized to the maximum value. X and Y: distance from the centre of the Langevin transducer along with respective direction.

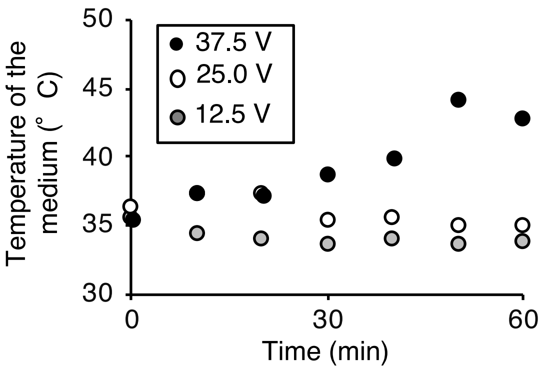

Figure S5. Time course of temperature changes over 1 h of ultrasonic exposure at each input voltage measured by a temperature sensor.
